# Conserved Non-Coding Sequences are Associated with Rates of mRNA Decay in *Arabidopsis*

**DOI:** 10.3389/fpls.2013.00129

**Published:** 2013-05-10

**Authors:** Jacob B. Spangler, Frank Alex Feltus

**Affiliations:** ^1^Department of Genetics and Biochemistry, Clemson UniversityClemson, SC, USA; ^2^Plant and Environmental Sciences, Clemson UniversityClemson, SC, USA

**Keywords:** conserved non-coding sequences, mRNA decay, polyploidy, gene regulation, *Arabidopsis*

## Abstract

Steady-state mRNA levels are tightly regulated through a combination of transcriptional and post-transcriptional control mechanisms. The discovery of *cis*-acting DNA elements that encode these control mechanisms is of high importance. We have investigated the influence of conserved non-coding sequences (CNSs), DNA patterns retained after an ancient whole genome duplication event, on the breadth of gene expression and the rates of mRNA decay in *Arabidopsis thaliana*. The absence of CNSs near α duplicate genes was associated with a decrease in breadth of gene expression and slower mRNA decay rates while the presence CNSs near α duplicates was associated with an increase in breadth of gene expression and faster mRNA decay rates. The observed difference in mRNA decay rate was fastest in genes with CNSs in both non-transcribed and transcribed regions, albeit through an unknown mechanism. This study supports the notion that some *Arabidopsis* CNSs regulate the steady-state mRNA levels through post-transcriptional control mechanisms and that CNSs also play a role in controlling the breadth of gene expression.

## Introduction

Duplication of genetic material has been proposed to be one of the primary evolutionary factors driving organism complexity and occurs at various scales ranging from single gene transpositions to whole genome duplication (WGD) events (Freeling and Thomas, 2006; Edger and Pires, 2009; Freeling, 2009; Schnable et al., 2011; Woodhouse et al., 2011). Instances of WGD are particularly prevalent in plants as roughly 35% of flowering plants are polyploid relative to their basal genera, and nearly all angiosperms have experienced an ancestral WGD (Sémon and Wolfe, 2007; Wood et al., 2009; Paterson et al., 2010; Jiao et al., 2011). Duplicate gene pairs that are retained post-duplication are expected to have either developed novel function (neofunctionalization) or distributed function between duplicated gene pairs (subfunctionalization) (Ohno, 1970; Force et al., 1999). The most likely outcome from a duplication event is the loss of additional genetic material through pseudogenization or deletion (fractionation) (Haldane, 1933; Nei and Roychoudhury, 1973; Freeling et al., 2012). However, many duplicated genes are enriched for particular biological functions (e.g., transcription factors, kinases, stress response), which suggests a more complex mechanism for gene retention (Blanc and Wolfe, 2004; Seoighe and Gehring, 2004; Zou et al., 2009).

The retention of specific functional classes encoded in duplicated genes suggests the fractionation process may involve a combination of factors including environmental cues, gene duplication scale (e.g., single gene transposition vs. WGD), and relative levels of gene expression (Birchler et al., 2005; Zou et al., 2009; Wang et al., 2011; Yang and Gaut, 2011). For instance, genes retained after a WGD event are thought to be retained more frequently relative to discrete duplication events as WGD events would copy all flanking DNA that encodes contains regulatory information (Schnable et al., 2011; Wang et al., 2011). Genes retained from WGD events in *Arabidopsis* and *Oryza* are consistent with this hypothesis, as they are less likely to display divergent expression patterns than duplicated genes from small-scale events (Casneuf et al., 2006; Wang et al., 2011). Through the study of conserved non-coding DNA sequence flanking duplicated loci (CNS elements), it is possible to identify specific regulatory motifs copied and retained after the duplication event.

*Arabidopsis thaliana* provides an excellent system to interpret the consequences of massive-scale gene duplication, as there have been three WGD events (Bowers et al., 2003; Maere et al., 2005; Barker et al., 2009). The most recent WGD in the *Arabidopsis* lineage was an ancient tetraploidy event that occurred roughly 23.2 Mya [α duplication event; (Bowers et al., 2003; Maere et al., 2005; Jiao et al., 2011)]. Remnants of the α event can be detected in the form of duplicate gene pairs (α duplicates) and CNS elements that have resisted fractionation (Thomas et al., 2007). Briefly, α duplicate CNS elements between 15 and 285 bp in length were discovered as local alignment high-scoring segment pairs between two α duplicate homeologs that did not overlap protein coding or transposon DNA.

The discovery of function encoded in CNS elements is an active area of research, as their discovery in *Arabidopsis* occurred within the last decade (Thomas et al., 2007). Recently, we identified a link between conserved non-coding sequences (CNSs) and the regulation of expression intensity, maintenance of co-expression between duplicate gene pairs, and association with known gene regulatory networks (Spangler et al., 2012a,b). Roughly half of the annotated CNSs contain known transcription factor binding sites (TFBS), although not all of the TFBS are functional (Freeling et al., 2007; Spangler et al., 2012a,b). We hypothesized that some intronic CNSs could be encoding intron-mediated enhancement (IME) regulatory mechanisms (Spangler et al., 2012b). Moreover, it was previously shown that CNSs were not related to small RNAs or transposable elements (Thomas et al., 2007). The contribution of CNSs to the regulation of gene expression is clear, but knowledge of the specific underlying regulatory mechanisms is incomplete.

While much focus on the regulation of mRNA levels has been at the transcriptional level, an increasing number of studies have focused on post-transcriptional control of steady-state mRNA levels (Shalem et al., 2008; Elkon et al., 2010; Vogel et al., 2010). The rates of mRNA degradation have been found to respond to various environmental and stress conditions, such as DNA damage, oxidative stress, and chemical exposure (Shalem et al., 2008; Elkon et al., 2010). Biological function also appears correlated with mRNA stability. Genes involved in metabolism tend to have longer half-lives, while regulatory genes tend to have shorter half-lives (Wang et al., 2002; Yang et al., 2003). Narsai et al. (2007) calculated the rates of decay for over 13,000 *Arabidopsis* genes and found the median half-life to be 3.8 h. While Narsai et al. focused on identifying DNA sequence elements in the 5′- and 3′- UTRs associated with mRNA decay rates, their analyses did not include gene duplication status or the presence of CNSs. Given the association of CNS position near α duplicates on predicted free folding energies of 5′-UTRs (Spangler et al., 2012b), we investigated any role of CNSs on mRNA stability.

The focus of this study was to examine potential post-transcriptional control of gene expression encoded in CNSs located near α duplicate gene coding sequences. We hypothesized that regulatory motifs encoded in some CNS elements control the steady-state mRNA levels in *Arabidopsis* at the level of RNA stability. We tested this hypothesis using the RNA decay information from Narsai et al., the most recent CNS annotation in *Arabidopsis*, and a collection of 7,158 publicly available microarray expression profiling datasets. We examined the effect of CNS gene position on the rate of mRNA decay and breadth of gene expression.

## Results

### Gene characteristics and mRNA decay rate

**Figure 1:**
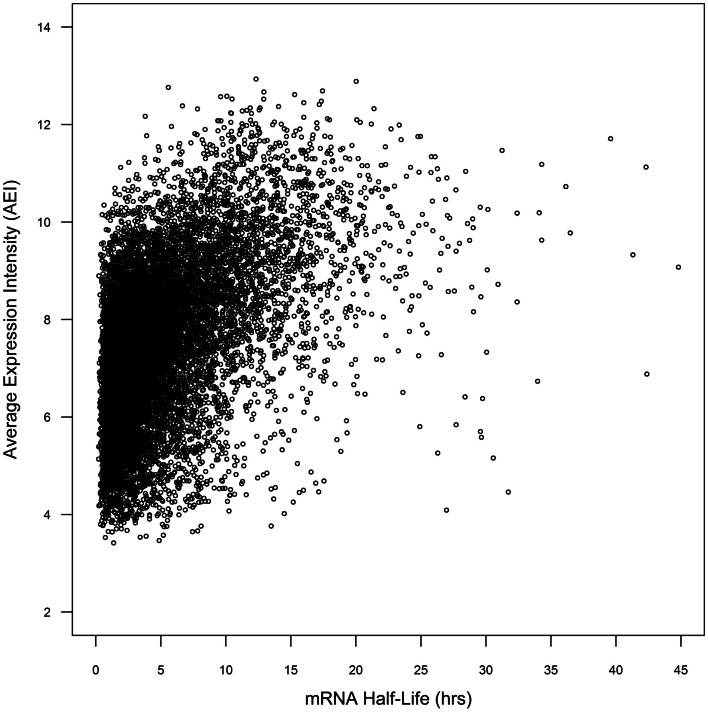
**Comparison of mRNA half-life vs. average expression intensity across genome**.

Whole genome duplicate gene pairs derived from the α duplication event (α duplicates) exhibit higher average levels of expression than other genes in *Arabidopsis* (Wang et al., 2011; Yang and Gaut, 2011). We had previously associated CNSs with changes in average expression intensity (AEI) and hypothesized that CNSs may influence mRNA stability (Spangler et al., 2012b). In a simple system, the steady-state mRNA concentration can be considered a combination of the rate of transcription and the rate of mRNA decay. We decided to test if the presence of CNSs was associated with changes in mRNA decay rates. To do this we collected the mRNA half-lives of 12,189 *Arabidopsis* genes from (Narsai et al., 2007). Within the 12,189 genes from Narsai et al. there was a significant correlation between AEI and mRNA half-life across 7,016 processed microarray datasets (Spearman’s rho = 0.462; *p* < 2.2 × 10^−16^; Figure 1), supporting the idea that AEI could be partially explained by the rate of mRNA decay.

**Figure 2:**
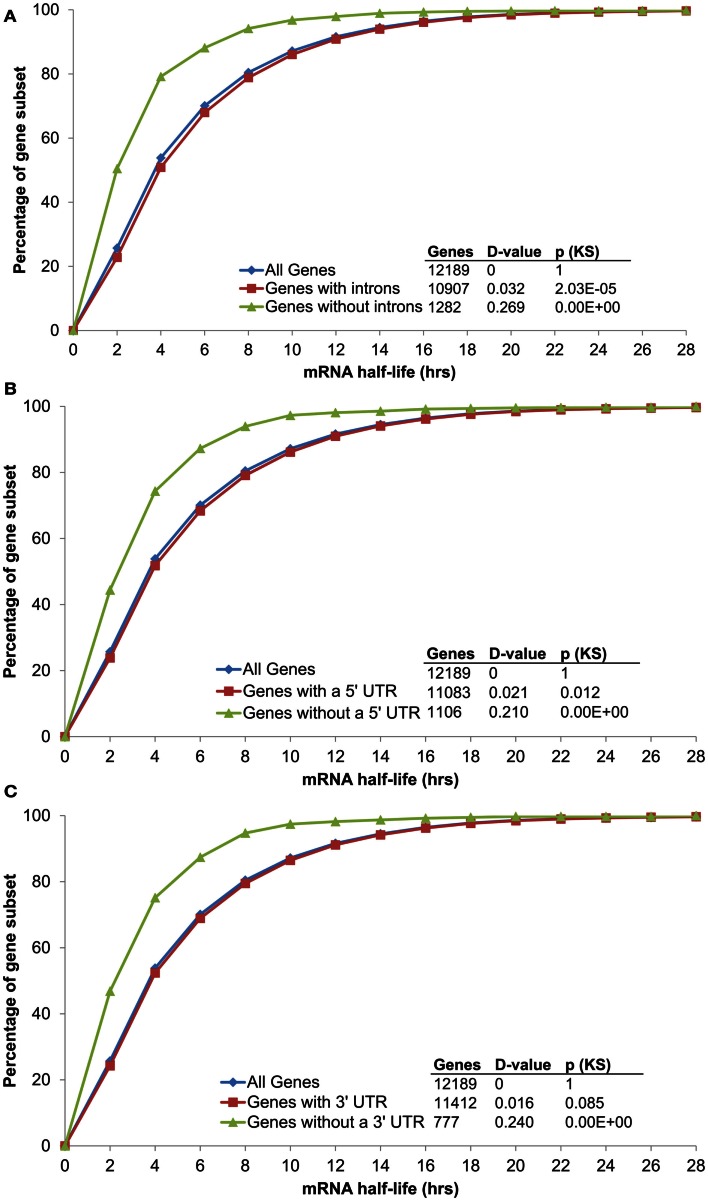
**The distribution of mRNA half-lives across genes grouped by (A) intron, (B) 5′ UTR, and (C) 3′ UTR annotation**.

Conserved non-coding sequences have been identified in all subgene positions relative to α duplicates [5′-upstream, 5′-UTR, intron, 3′-UTR, and 3′-downstream (Thomas et al., 2007; Spangler et al., 2012b)]. While only ∼34% of CNSs are located within transcribed subgene positions (5′-UTR, intron, and 3′-UTR), each of these regions have been associated with changes in mRNA stability independent of CNS annotation (Decker and Parker, 1993; Peng et al., 1998; Lindquist et al., 2004; Meng et al., 2005; Wang et al., 2005; Narsai et al., 2007). For example, Narsai et al. identified that the absence of an intron was sufficient to decrease mRNA half-life (Narsai et al., 2007) and this pattern was maintained with updated *Arabidopsis* annotation [TAIR10; Figure 2A; Kolmogorov–Smirnov *p*-value (KS-*p*) test *p* = <2.20 × 10^−16^]. Notably, the absence of an annotated 5′-UTR or 3′-UTR was also sufficient to decrease mRNA stability (Figures 2B,C; KS-*p* = <2.20 × 10^−16^ and <2.20 × 10^−16^, respectively). With the objective of identifying changes in mRNA that could be attributed to CNS presence, we therefore limited our analyses to the 9,958 genes measured by Narsai et al. that contained annotated 5′-UTR, intron, and 3′-UTR sequences. The list of 9,958 genes was separated into the three categories based on gene duplication status: α duplicates, singletons, and non-α duplicates. We considered a *p*-value ≤ 0.001 significant for all comparisons.

### CNS presence and mRNA decay rate

**Figure 3:**
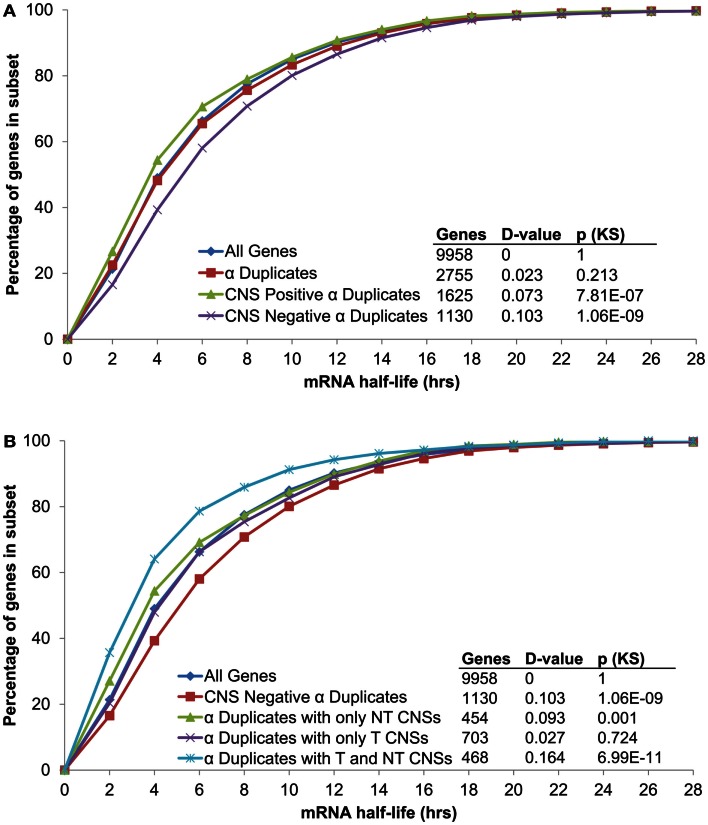
**The distribution of mRNA half-lives across α duplicates grouped by (A) CNS presence/absence and (B) subgene position restricted CNS annotation**. Each gene subset is restricted to genes with annotated 5′ UTR, intron, and 3′ UTR sequence. The *D*-value represents the distance between the distributions and was used in the Kolmogorov–Smirnov (KS) statistic to determine statistical difference. CNS, conserved non-coding sequence; NT, non-transcribed; T, transcribed.

In order to examine if CNSs alter the rate of mRNA decay we separated α duplicates into two gene subsets based on CNS presence. We found CNS negative α duplicates (α duplicates with no CNSs) had an increased mRNA half-life relative to all genes (median 5.02 and 4.11 h, respectively; KS-*p* = 1.06 × 10^−9^; Figure 3A; Table 1). Notably, CNS positive α duplicates (α duplicates with at least one CNS) had a decreased mRNA half-life relative to all genes (median 3.57 and 4.11 h, respectively; KS-*p* = 7.81 × 10^−7^; Figure 3A; Table 1). The difference in mRNA half-life between CNS positive α duplicates and CNS negative α duplicates was also significant (median 3.57 and 5.02 h, respectively; KS-*p* = 5.33 × 10^−15^; Table 1; Table S1 in Supplementary Material).

**Table 1:** **Gene expression characteristics of *Arabidopsis* gene subsets**.

Gene subset	Genes	mRNA HL	τ	CV
All genes	9958	4.11	0.287	0.099
α Duplicates	2755	4.21	0.289	0.102
Singleton	2092	4.35	0.277	0.093
Non-α duplicates	5111	3.96	0.290	0.101
CNS negative α duplicates	1130	5.02	0.281	0.099
CNS positive α duplicates	1625	3.57*	0.296*	0.103
α Duplicates with only NT CNSs	454	3.51*	0.304*	0.110*
α Duplicates with only T CNSs	703	4.24*	0.283	0.094*
α Duplicates with T and NT CNSs	468	2.85*	0.319*	0.114*

*p-Value < 0.001 via KS test compared to CNS negative α duplicates.

*All values shown for mRNA HL, τ and CV are medians; T, transcribed; CV, coefficient of variation; HL, mRNA half-life (hrs); NT, non-transcribed*.

As AEI can vary based on CNS subgene position, we looked for a similar effect on the rate of mRNA decay by examining the half-lives of α duplicates with only non-transcribed CNSs, α duplicates with only transcribed CNSs, and α duplicates with both non-transcribed and transcribed CNSs. There was no difference in mRNA half-life for α duplicates with only non-transcribed CNSs relative to all genes (median 3.51 and 4.11 h, respectively; KS-*p* = 1.03 × 10^−3^; Figure 3B; Table 1). The mRNA half-life for α duplicates with only non-transcribed CNSs was significantly lower relative to CNS negative α duplicates (3.51 and 5.02 h, respectively; KS-*p* = 2.92 × 10^−8^; Table 1; Table S1 in Supplementary Material). No significant change was observed in mRNA half-life between α duplicates with only transcribed CNSs relative to all genes (median 4.24 and 4.11 h, respectively; KS-*p* = 0.72; Figure 3B; Table 1), although the mRNA half-life for α duplicates with only transcribed was lower than CNS negative α duplicates (median 4.24 and 5.02 h, respectively; KS-*p* = 8.74 × 10^−4^; Table 1; Table S1 in Supplementary Material). Interestingly, there was a significant decrease in mRNA half-life for α duplicates with both non-transcribed and transcribed CNSs compared to all genes (median 2.85 and 4.11 h, respectively; KS-*p* = 6.99 × 10^−11^; Figure 3B; Table 1) and this decrease in mRNA half-life for α duplicates with both non-transcribed and transcribed CNSs was also lower than CNS negative α duplicates (2.85 and 5.02 h, respectively; KS-*p* < 2.20 × 10^−16^; Table 1; Table S1 in Supplementary Material). All pairwise comparisons for mRNA half-life were also made using Wilcoxon ranked sum tests and resulted in similar patterns of significance (Table S2 in Supplementary Material).

**Figure 4:**
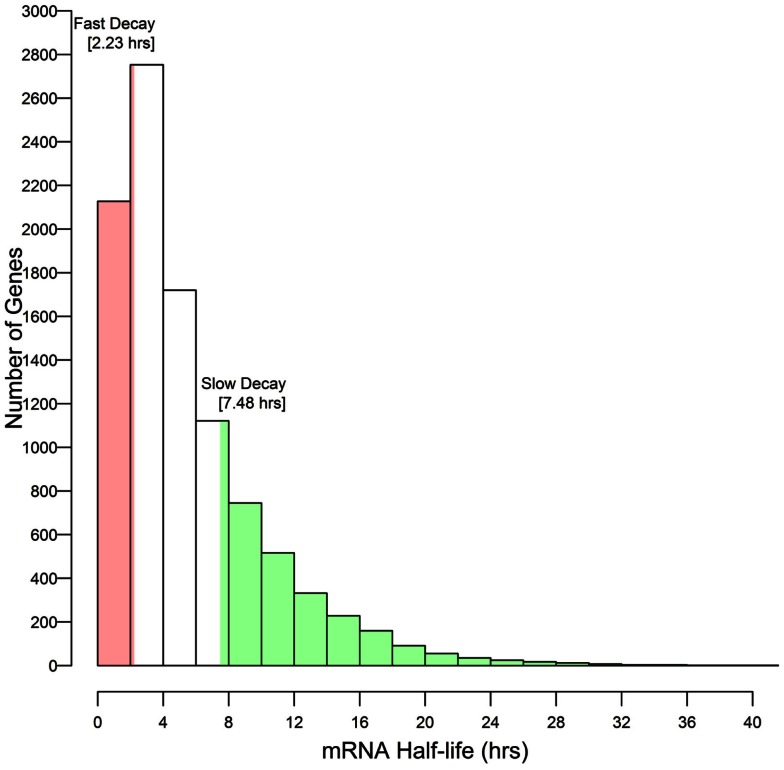
**Selection of outlier genes with fast and slow rates of mRNA decay relative to the distribution of mRNA half-lives**.

These results associate CNS annotation with an increase (CNS positive α duplicates) or decrease (CNS negative α duplicates) in rate of mRNA decay relative to genomic background. In order to verify this trend using a reverse approach we isolated the genes with the fastest rates of mRNA decay (lower quartile; ≤2.23 h) and genes with the slowest rates of mRNA decay (upper quartile; ≥7.48 h) and looked for enrichment or depletion of CNS annotation (Figure 4). Genes with the fastest rates of mRNA decay were enriched in CNS positive α duplicates relative to the genomic background (20.0 vs. 16.3%, respectively; Fisher’s *p*-value (FI-*p*) = 2.05 × 10^−5^). Notably, genes with the fastest rates of mRNA decay were also depleted in CNS negative α duplicates relative to the genomic background (8.6 vs. 11.4%, respectively; FI-*p* = 7.12 × 10^−5^). Genes with the slowest rates of mRNA decay were enriched in CNS negative α duplicates relative to background (14.3 vs. 11.4%, respectively; FI-*p* = 8.08 × 10^−5^). Genes with the slowest rates of mRNA decay had no change in the proportion of CNS positive α duplicates relative to background (15.1 vs. 16.3%, respectively; FI-*p* = 0.14).

### CNS presence and breadth of gene expression

As mentioned previously, a simple model of steady-state mRNA levels (e.g., AEI) could be explained by the combination of transcriptional rate and mRNA decay. Since we observed significant differences in mRNA half-life between CNS positive α duplicates and CNS negative α duplicates, we therefore hypothesized that any variance of gene expression across the microarray datasets could be partially regulated by CNSs through an mRNA decay mechanism. To determine if the observed changes in mRNA decay based on CNS annotation could be attributed to broad (many tissues or conditions) or narrow (few tissues or conditions) gene expression, we examined the sample variance of expression intensity for all genes across the 7,016 expression datasets. We selected the metric τ to quantify the sample variance, as it is similar to the coefficient of variation (CV), but has been reported to be superior compared to CV for measuring breadth of gene expression (Liao and Zhang, 2006). A τ = 1 represents expression in only a single microarray experiment, while a τ = 0 represents expression across all 7,016 microarray experiments in our study.

**Figure 5:**
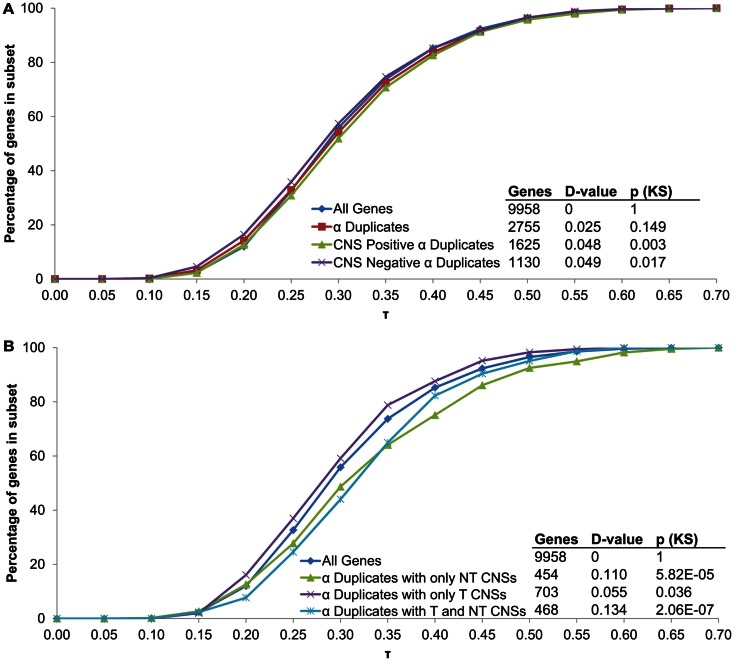
**The distribution of τ across α duplicates grouped by (A) CNS presence/absence and (B) subgene position restricted CNS annotation**. Each gene subset is restricted to genes with annotated 5′ UTR, intron, and 3′ UTR sequence. The *D*-value represents the distance between the distributions and was used in the Kolmogorov–Smirnov (KS) statistic to determine statistical difference. CNS, conserved non-coding sequence; NT, non-transcribed; T, transcribed.

All α duplicates were then dissected into two gene subsets based on CNS presence. Unlike rates of mRNA decay, there was no difference in τ for CNS negative α duplicates relative to all genes (median 0.281 and 0.287, respectively; KS-*p* = 0.02; Figure 5A; Table 1). Similarly, CNS positive α duplicates also had no difference in τ relative to all genes (median 0.296 and 0.287, respectively; KS-*p* = 3.11 × 10^−3^; Figure 5A; Table 1). Markedly, CNS positive α duplicates had significantly higher τ (narrower expression) than CNS negative α duplicates (median 0. 296 and 0.281, respectively; KS-*p* = 7.14 × 10^−4^; Table 1; Table S1 in Supplementary Material).

We then examined α duplicates separated into gene subsets based on CNS subgene position. There was a significant increase in τ (narrower expression) for α duplicates with only non-transcribed CNSs relative to all genes (median 0.304 and 0.287, respectively; KS-*p* = 5.82 × 10^−5^; Figure 5B; Table 1). The increase in τ for α duplicates with only non-transcribed CNSs was also significant relative to CNS negative α duplicates (median 0.304 and 0.281, respectively; KS-*p* = 3.74 × 10^−4^; Table 1; Table S1 in Supplementary Material). There was no difference in τ for α duplicates with only transcribed CNSs relative to all genes (median 0.283 and 0.287, respectively; KS-*p* = 0.04; Table 1). Additionally, α duplicates with only transcribed CNSs had no change in τ relative to CNS negative α duplicates (median 0.283 and 0.281, respectively; KS-*p* = 0.19; Figure 5B; Table 1; Table S1 in Supplementary Material). Interestingly, there was an increase in τ (narrower expression) for α duplicates with both non-transcribed and transcribed CNSs relative to all genes (median 0.319 and 0.287, respectively; KS-*p* = 2.06 × 10^−7^; Figure 5B; Table 1). The increase in τ for α duplicates with both non-transcribed and transcribed CNSs was also significant relative to CNS negative α duplicates (median 0.319 and 0.281, respectively; KS-*p* = 1.85 × 10^−7^; Table 1; Table S1 in Supplementary Material). All pairwise comparisons for τ were also made using Wilcoxon ranked sum tests and resulted in similar patterns of significance (Table S2 in Supplementary Material).

### CNS’ annotation and gene expression characteristics

The initial screen of CNS elements was limited to α duplicate pairs (Thomas et al., 2007). However, there is the possibility that CNS elements exist elsewhere in the genome near singletons, non-α duplicates or in non-duplicated form surrounding other α duplicates. We had identified additional CNS elements throughout the *Arabidopsis* genome and labeled these elements as CNS’ (Spangler et al., 2012a). We tested for differences in mRNA half-life, τ, and CV across α duplicates, singletons, and non-α duplicates with and without CNS’ annotation. As per the CNS analysis, we found that CNS’ positive α duplicates had significantly shorter mRNA half-lives than CNS’ negative α duplicates (median 3.95 and 5.29 h, respectively; KS-*p* = 8.31 × 10^−8^; Table 2; Table S3 in Supplementary Material). Similar to the CNS-only analysis, there was no significant difference between CNS’ positive α duplicates and CNS’ negative α duplicates for τ (median 0.291 and 0.284, respectively; KS-*p* = 0.29; Table 2; Table S3 in Supplementary Material). Interestingly, there was no difference in mRNA half-life between CNS’ positive singletons and CNS’ negative singletons (median 4.21 and 4.59 h, respectively; KS-*p* = 0.10; Table 2; Table S3 in Supplementary Material). There was also no difference in mRNA half-life between CNS’ positive non-α duplicates and CNS’ negative non-α duplicates (median 3.95 and 3.98 h, respectively; KS-*p* = 0.94; Table 2; Table S3 in Supplementary Material). All pairwise comparisons for CNS’ gene subsets were also made using Wilcoxon ranked sum tests and resulted in similar patterns of significance (Table S4 in Supplementary Material).

**Table 2:** **Gene expression characteristics based on CNS’ annotation**.

Gene subset	Genes	mRNA HL	τ	CV
CNS’ negative α duplicates	497	5.29	0.284	0.101
CNS’ positive α duplicates	2258	3.95*	0.291	0.102
CNS’ negative singletons	940	4.59	0.274	0.092
CNS’ positive singletons	1152	4.21	0.278	0.094
CNS’ negative Non-α duplicates	2202	3.98	0.287	0.100
CNS’ positive Non-α duplicates	2909	3.95	0.291	0.103

*p-Value < 0.001 via KS test compared to CNS’ negative α duplicates.

*All values shown for mRNA HL, τ and CV are medians*.

*CV, coefficient of variation; HL, mRNA half-life (hrs)*.

## Discussion

While the ability of CNSs to influence steady-state mRNA levels at the transcriptional level has previously been examined, the potential for post-transcriptional regulation by CNSs was limited to examining IME and predicted 5′-UTR folding energies (Spangler et al., 2012b). In this study, we associated the presence of CNSs with faster rates of mRNA decay and the absence of CNSs with slower rates of mRNA decay. We suggest these differences in rates of mRNA decay are partially responsible for changes in breadth of gene expression (τ and CV). Broadly, this study and previous results supports our working hypothesis that CNSs encode multiple regulatory mechanisms and influence steady-state mRNA levels at both transcriptional and post-transcriptional levels.

Within this study we found the presence of CNSs was sufficient to significantly reduce mRNA half-life by ∼0.5 h relative to all genes and ∼1.5 h relative to CNS negative α duplicates (Table 1). This reduction in mRNA stability was further supported by the enrichment of CNS positive α duplicates within genes with the fastest rates of mRNA decay. The reduction in mRNA half-life appeared to be partially dependent on CNS subgene position, as α duplicates with only transcribed CNSs were the most similar to the genomic background and had the smallest difference in mRNA half-life relative to CNS negative α duplicates. Additionally, there was no correlation between CNS frequency and rate of mRNA decay for α duplicates with only transcribed CNSs (Spearman’s rho = −0.08; *p* = 0.02) or α duplicates with only non-transcribed CNSs (Spearman’s rho = −0.09; *p* = 0.05). This suggests that the presence of even a single non-transcribed CNS may be sufficient to reduce mRNA half-life. We attempted to narrow the effect of CNSs on mRNA half-life to individual subgene positions (e.g., 5′-upstream, 5′-UTR), but were unable to detect any significant differences (data not shown).

The association of non-transcribed CNSs (5′-upstream and 3′-downstream) with an increased rate of mRNA decay is a surprising finding given that any RNA decay motifs encoded in the CNS would not be present in the preprocessed or mature RNA transcript. The mechanism by which non-transcribed CNSs are influencing the rate of mRNA decay is unknown, but non-transcribed CNSs are in phase with increased mRNA decay. It may be that α duplicates with non-transcribed CNS are associated with motifs that are not encoded within the CNS. For example, a number of genes in *Arabidopsis* contain miRNA target motifs within their coding regions (Llave et al., 2002; Rhoades et al., 2002; Chen, 2004), and some genes contain coding region motifs recognized by RNA binding proteins that reduce transcript stability (Chang et al., 2004; Lee and Gorospe, 2011). The potential for α duplicates to contain novel *cis*-regulatory post-transcriptional motifs within their coding sequence is interesting and should be considered in future studies. It is possible that the CNS is coupled to a conserved coding (i.e., CDS) motif that would be bypassed by the way CNSs were discovered.

**Figure 6:**
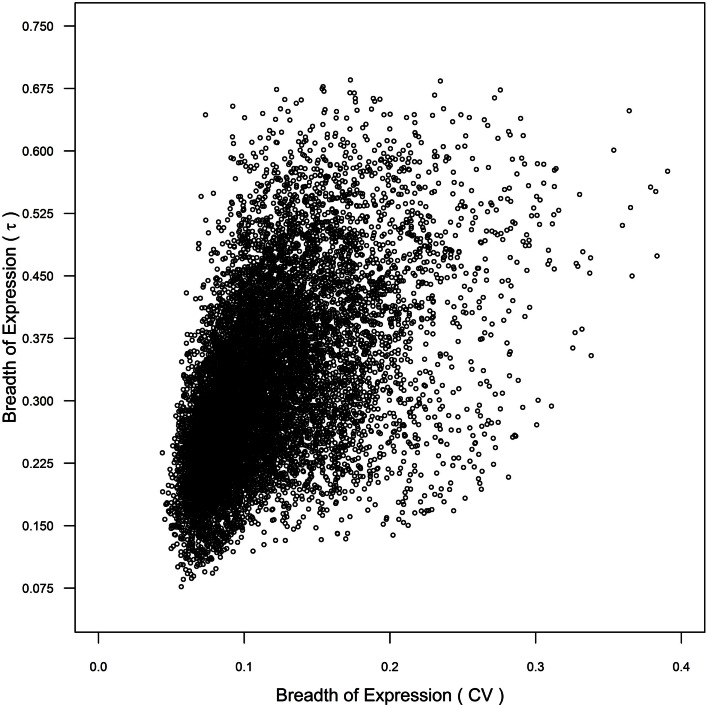
**Comparison of breadth of gene expression as measured by coefficient of variance (CV) and τ across genome**.

α Duplicates with non-transcribed CNSs and α duplicates with both non-transcribed and transcribed CNSs demonstrate narrower expression (higher τ) than CNS negative α duplicates, which suggests that non-transcribed CNSs may contain *cis*-regulatory elements responsible for controlling breadth of gene expression. However, only α duplicates with both non-transcribed and transcribed CNSs had lower mRNA half-lives than CNS negative α duplicates, suggesting that the changes in breadth expression are only partially regulated at the level of mRNA decay. The differences in breadth of expression between the gene subsets we tested were also maintained using CV as our metric of breadth of gene expression, although the statistical differences were less defined than τ (Tables S1–S4 in Supplementary Material). The similarity between metrics was due, in part, to a correlation between CV and τ (Spearman’s rho = 0.556; *p* < 2.20 × 10^−16^; Figure 6). These results further support that τ provides an improved level of resolution for measuring breadth of gene expression, and that CNSs assist in the control breadth of gene expression.

**Figure 7:**
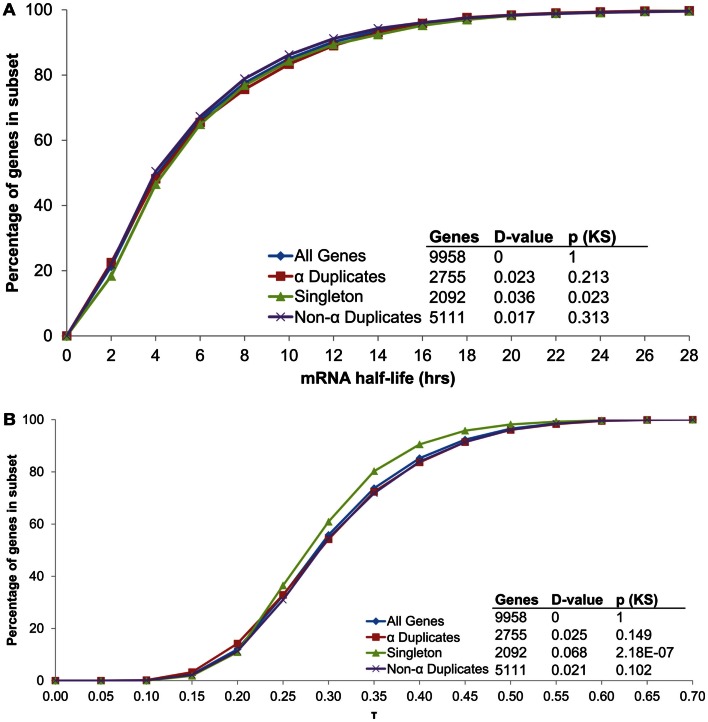
**The distribution of (A) mRNA half-lives and (B) τ across genes grouped by duplication status**.

Although α duplicates have higher expression level (AEI) relative to other genes in *Arabidopsis* (Wang et al., 2011; Yang and Gaut, 2011), we found α duplicates to only have a small increase in AEI relative to all genes within our dataset (median 7.79 and 7.73, respectively; KS-*p* = 2.82 × 10^−4^). The small differences in AEI were also reflected in mRNA half-life as we found no significant differences in mRNA half-life between α duplicates, singletons and non-α duplicates relative to all genes (Figure 7A). Intriguingly, we did observe a difference in AEI between CNS positive α duplicates and CNS negative α duplicates (median 7.68 and 7.94, respectively; KS-*p* = 5.09 × 10^−4^), further supporting a link between AEI and mRNA half-life. While there was no effect of gene duplication status on mRNA half-life, we did observe a significant decrease in τ for singletons relative to all genes (Figure 7B). This had been previously observed in *Arabidopsis* (Yang and Gaut, 2011) and supports the hypothesis that mRNA stability only partially controls the breadth of gene expression.

Expanding our analysis to CNS elements outside of α duplicate gene pairs (CNS’), it was found that there was still a significant difference in mRNA decay between CNS’ positive α duplicates relative to CNS’ negative α duplicates (Table 2). However, CNS’ presence did not have any detectable influence on mRNA half-life for singletons or non-α duplicates. We propose the following hypotheses regarding these observations: (i) the DNA sequence in CNS’ elements has diverged sufficiently or lost appropriate positional proximity that post-transcriptional regulation was lost; (ii) CNS elements must be maintained in duplicate form for post-transcriptional regulation to function correctly; (iii) CNS’ elements are false positive *cis*-regulatory motifs. There is evidence to dispute the third hypothesis, as CNS’ elements have been found to overlap with known gene regulatory networks (Spangler et al., 2012a). Further research on CNS’ elements would help to test these hypotheses.

Rates of mRNA decay have been correlated with several functional classes of genes, such as kinases, plasma membrane proteins and transcription factors (Wang et al., 2002; Yang et al., 2003; Narsai et al., 2007). Notably, α duplicates are enriched in some of these functional classes [e.g., transcription factors; (Thomas et al., 2007)]. In addition, rates of mRNA decay are known to vary based on various environmental stimuli, such as chemical exposure, oxidative stress, or DNA damage (Shalem et al., 2008; Elkon et al., 2010), which would depend on regulatory signals such as transcription factors. However, upon examination of each CNS gene subset there was no significant enrichment of functional terms (e.g., GO, KEGG) beyond annotation previously associated with α duplicates [e.g., transcription factors, kinases; Table S5 in Supplementary Material; (Blanc and Wolfe, 2004; Seoighe and Gehring, 2004; Thomas et al., 2007)]. Therefore this suggests that the differences in mRNA stability associated with CNS presence or absence cannot be attributed to an obvious functional class.

Our working hypothesis is that CNSs are *cis*-regulatory DNA elements that influence mRNA steady-state levels, and the regulatory mechanisms encoded in the CNSs are a combination of transcriptional and post-transcriptional control. The prevailing hypothesis for the fractionation bias observed after most WGD events is that genes more sensitive to variation in dosage, possibly conferred by CNS encoded regulation, have a higher impact on fitness and are more likely to be retained in duplicated gene pairs (Birchler and Veitia, 2007; Schnable et al., 2012). In this case, the organism’s ability to tightly regulate gene dosage via an mRNA decay mechanism after a WGD event would provide a selective advantage. More specifically, within this study we provide evidence that post-transcriptional control of α duplicate pairs could be mediated through CNSs via mRNA decay mechanisms. We have included the list of genes with CNS sequence and mRNA decay rate for further testing of this hypothesis at the individual gene level (Table S6 in Supplementary Material). Although CNSs are only one component of the complete regulation story, genes with CNSs are more likely to be maintained across multiple WGD events (Schnable et al., 2011, 2012), and it may be that the regulatory flexibility conferred by CNSs to regulate gene dosage has played an integral role to the retention of many α duplicates following the α WGD event.

## Materials and Methods

### Identification of gene duplication status

The list of α duplicates gene pairs were collected from (Thomas et al., 2006) and were updated to TAIR10 annotation, reducing the list of 3,166 gene pairs to 3,118. Genes with only self BLASTP hits (E < 10^−10^) in the TAIR10 genome were considered singletons. There were 5,108 genes that met this criterion in the TAIR10 genome. Any gene that was not an α duplicate or singleton was assigned to the category of non-α duplicates.

### Microarray collection and genome annotation

A total of 7,158 *Arabidopsis* ATH1 Genome Array experiments were obtained from NCBI GEO (platform GPL198). RMA normalization (Irizarry et al., 2003) was performed for all samples together using the command-line utility of RMAExpress^1^. Sample outlier detection was performed using the arrayQualityMetrics (Kauffmann et al., 2009) tool for Bioconductor (Gentleman et al., 2004). Samples that failed two of the three outlier tests were removed from the dataset. The remaining dataset consisted of 7,016 microarray experiments. All probe sets were then mapped to genes using ATH1 mappings available via TAIR (Swarbreck et al., 2008)^2^. Of the original 22,810 probe sets on the ATH1 platform, all Affymetrix control probe sets (prefixed with AFFX), probe sets that did not map to a gene model in TAIR10 (non-genic), or probe sets that mapped to multiple loci (ambiguous) were removed. The final count of probe sets used was 21,107. Any values calculated for probe sets that were shared by a single gene (redundant) were averaged. The list of CEL files used can be found in Table S7 in Supplementary Material.

### mRNA stability estimates

Observed mRNA half-lives were collected from the supplementary information of (Narsai et al., 2007) and included data for 13,012 probe sets. The probe sets were reduced to exclude non-genic and ambiguous probe sets. The final count of probe sets analyzed was 12,327. Half-lives for probe sets that were shared by a single gene (redundant) were averaged and resulted in 12,189 genes. The distributions of mRNA half-life were compared using the Kolmogorov–Smirnov test (KS test) and Wilcoxon ranked sum test (Wilcox test) in *R*. The associated *p*-values can be found in Tables S1–S4 in Supplementary Material.

### Breadth of gene expression

The breadth of gene expression was measured with the index τ (Yanai et al., 2005; Yang and Gaut, 2011):

$$\tau =\frac{\overset{n}{\underset{j=1}{\sum }}\left[1-\frac{\log_{2}S\left(i,j\right)}{\log_{2}S\left(i,\max\right)}\right]}{n-1}$$

*S*(*i*, max) represents the maximum expression intensity for the given probe set across all microarray experiments. Genes with a τ = 0 represent expression across all microarrays, while genes expressed in only one microarray will approach τ = 1. Breadth of gene expression was also measured using the coefficient of variation (CV = σ/μ) for each probe set.

### Functional enrichment within CNS subgene position exclusive α duplicates

α Duplicates were separated into CNS positive α duplicates, CNS negative α duplicates, α duplicates with only non-transcribed CNSs, α duplicates with only transcribed CNSs, and α duplicates with both non-transcribed and transcribed CNSs. These gene lists were then tested for enrichment of functional terms using a DAVID-like (Huang et al., 2007) functional profiling strategy using in-house Perl scripts (Huang et al., 2008; Ficklin et al., 2010). All terms were tested for enrichment across each gene list via a Fisher’s exact test using a Perl script. Any terms with a Bonferroni *p* ≤ 0.001 were considered significantly enriched. All GO^3^ and Interpro^4^ annotations were downloaded from TAIR. All TAIR10 peptide sequences (TAIR10_pep_20101214.txt) were downloaded from^5^ and submitted to the KEGG Automatic Annotation server on 10-26-2011 (Moriya et al., 2007). All Pfam domains were obtained from the Sanger database^6^. Enrichment of functional terms including gene ontology (GO), protein domains (Interpro and Pfam) and biochemical pathways (KEGG) can be found in Table S5 in Supplementary Material.

### CNS annotation

All CNS annotation was collected from the supplemental data of (Spangler et al., 2012b).

### CNS’ annotation

All CNS’ annotation was collected from the supplemental data of (Spangler et al., 2012a). The associated *p*-values from all Kolmogorov–Smirnov test and Wilcoxon ranked sum tests with CNS’ can be found in Tables S3 and S4 in Supplementary Material.

### TAIR10 UTR annotation

All TAIR10 5′-UTR, intron, and 3′-UTR sequences were downloaded from TAIR (TAIR10_5_utr_20101028, TAIR10_intron_20101028 and TAIR10_3_utr_20101028).

## Conflict of Interest Statement

The authors declare that the research was conducted in the absence of any commercial or financial relationships that could be construed as a potential conflict of interest.

## Supplementary Material

The Supplementary Material for this article can be found online at http://www.frontiersin.org/Plant_Genetics_and_Genomics/10.3389/fpls.2013.00129/abstract

Supplementary Table S1 **KS Test *p*-values for CNS Gene Subsets**.

Supplementary Table S2 **Wilcoxon Ranked Sum *p*-values for CNS Gene Subsets**.

Supplementary Table S3 **KS Test *p*-values for CNS’ Gene Subsets**.

Supplementary Table S4 **Wilcoxon Ranked Sum *p*-values for CNS’ Gene Subsets**.

Supplementary Table S5 **Enriched Function Annotation in Alpha Duplicate Gene Lists based on CNS Annotation (Bonferroni *p*-value ≤ 0.001)**.

Supplementary Table S6 **Arabidopsis Gene mRNA Half-Life and CNS Status**.

Supplementary Table S7 **GEO Datasets Used in This Study**.

## Footnotes

^1^http://rmaexpress.bmbolstad.com/

^2^affy_ATH1_array_elements-2010-12-20.txt; ftp://ftp.arabidopsis.org/home/tair/Microarrays/Affymetrix/

^3^ATH_GO_GOSLIM.txt; ftp://ftp.arabidopsis.org/home/tair/Ontologies/Gene_Ontology; 10-25-2011

^4^TAIR10_all.domains; ftp://ftp.arabidopsis.org/home/tair/home/tair/Proteins/Domains/; 11-18-2010

^5^ftp://ftp.arabidopsis.org/home/tair/Proteins/TAIR10_protein_lists

^6^ftp://ftp.sanger.ac.uk/pub/databases/Pfam/current_release/database_files/pfamA.txt.gz
